# Supplementary material for: Low CDK Activity and Enhanced Degradation by APC/CCDH1 Abolishes CtIP Activity and Alt-EJ in Quiescent Cells
Source: Cells. 2023 Jun 1;12(11):1530. doi: 10.3390/cells12111530 (PMC10252496; doi:10.3390/cells12111530)
Supplement: Supplementary file 1 [file cells-12-01530-s001.zip › cells-2320445-supplementary.pdf]

Supplementary documents

Low CDK activity and enhanced degradation by APC/C^{CDH1} suppress CtIP activity and alt-EJ in quiescent cells

Fanghua Li, Emil Mladenov, Yanjie Sun, Aashish Soni, Martin Stuschke, Beate Timmermann and George Iliakis

Table S1. Sequence of siRNAs and gRNAs

Name	Sequence
siRNA targeting CTIP:	GCUAAAACAGGAACGAAUC [1]
siRNA targeting FZR1/CDH1:	GGAUUAACGAGAAUGAGAA [2]
siRNA targeting EXO1:	UGCCUUUGCUAAUCCAAUCCCACGC [3]
siRNA targeting DNA2:	AGACAAGGUUCCAGCGCCA [4]
negative control siRNA:	UUCUCCGAACGUGUCACGU [5]
gRNA targeting *POLQ* (Sense)	TGAATCTTCTGCGTCGGAGT
gRNA targeting *POLQ* (anti-sense)	AAACTCTTCTCGGCCGATCA

Table S2. Inhibitors

Name	Provider	Target and working concentration
8-(4-Dibenzothienyl)-2-(4-morpholinyl)-4H-1-benzopyran-4-one: **Nu7441**	Tocris Bioscience	DNA-PKcs, 2.5µM
6-acetyl-8-cyclopentyl-5-methyl-2-[[5-(1-piperazinyl)-2-pyridinyl] amino]pyrido[2,3-d]pyrimidin-7(8H)-one 2-hydroxy-ethanesulfonic acid: **PD0332991**	Selleckchem	CDK4/6, 500 nM
2,2'-[[6-[[(4-Methoxyphenyl) methyl]amino]-9-(1-methylethyl)-9H-purin-2-yl]imino]bis[ethanol]: **CVT313**	Selleckchem	CDK2, 5 µM
5-(6-Quinolinylmethylene)-2-((2-thienylmethyl)amino)-4(5H)-thiazolone: **RO3306**	Selleckchem	CDK1, 10 µM
2-Amino-5-[(4-hydroxyphenyl)methylene]-4(5H)-thiazolone: **Mirin**	Santa Cruz Biotechnology	MRE11 exonuclease, 50 µM
(5*Z*)-5-[(4-Hydroxyphenyl)methylene]-3-(2-methylpropyl)-2-thioxo-4-thiazolidinone: **PFM01**	Selleckchem	MRE11 endonuclease, 50 µM
5-(5,6-Dimethoxy-1*H*-benzimidazol-1-yl)-3-[[2-(trifluoromethyl)phenyl]methoxy]-2-thiophenecarboxamide: **GW843682X**	Tocris Bioscience	PLK1/3, 2µM
(1R)-3-Methyl-1-({(2S)-3-phenyl-2-[(2-pyrazinylcarbonyl)-amino]propanoyl} amino)butylboronsäure: **Bortezomib**	Selleckchem	Proteasome, 2µM

Table S3. Antibodies

Name	Provider	Cat#
Mouse monoclonal anti-RPA70B	Dr. J. Hurwitz [6]	N/A
Mouse monoclonal anti-BrdU	BD Biosciences	Cat# 347580
Rabbit polyclonal anti-POLΘ	ABCAM	Cat# ab80906
Rabbit monoclonal anti-CTIP	Cell Signaling Technonology	Cat# 9201
Mouse monoclonal anti-FZR/CDH1	Santa Cruz Biotechnology	Cat# sc-56312
Mouse monoclonal anti-CCNB1	Santa Cruz Biotechnology	Cat# sc-245
Rabbit polyclonal anti-CCNA2	Santa Cruz Biotechnology	Cat# sc-596

Rabbit polyclonal anti-CCND1	Cell Signaling Technonology	Cat# 2922
Mouse monoclonal anti-CCNE1	Cell Signaling Technonology	Cat# 4129
Rabbit polyclonal anti-Ki67	ABCAM	Cat# ab15580
Rabbit polyclonal anti-MRE11	Novus Biologicals	Cat# NB100-142
Mouse monoclonal anti-NBS1/NIBRIN	Santa Cruz Biotechnology	Cat# sc-374168
Mouse monoclonal anti-RAD50	GeneTex	Cat# GTX70228
Rabbit polyclonal anti-CtIP-pT847	This paper	N/A
Rabbit polyclonal anti-EXO1	GeneTex	Cat# GTX109891
Rabbit polyclonal anti-DNA2	Proteintech	Cat# 18727-1-AP
Mouse monoclonal anti-γH2AX	GeneTex	Cat# GTX628789
Mouse monoclonal anti-GAPDH	UbpBio	Cat# Y1041
Mouse monoclonal anti-RAD51	Gene Tex	Cat# GTX70230
Mouse monoclonal anti-RB1	BD Biosciences	Cat#554145
Rabbit monoclonal anti-RB1-pS807/811	Cell signaling Technology	Cat#9308
Rabbit monoclonal anti-β-Actin	UbpBio	Cat# Y1055
Alexa Fluor 488 Goat anti-Mouse IgG (H+L)	Thermo Fisher Scientific	Cat# A11001
IRDye 680RD Goat anti-Mouse IgG (H+L)	LI-COR Biosciences	Cat#926-68020
IRDye 800CW Goat anti-Mouse IgG (H+L)	LI-COR Biosciences	Cat#926-32210
IRDye 680RD Goat anti-Rabbit IgG (H+L)	LI-COR Biosciences	Cat#926-68021
IRDye 800CW Goat anti-Rabbit IgG (H+L)	LI-COR Biosciences	Cat#926-32211

Table S4. Primers for real time-PCR

**Name**	**Sequence**
CtIP-Fwd	AGAAGATCGGTTAAGAGCAGGC
CtIP-Rev	TGCTGGAGTTGTTCAGAAAGC
GAPDH-Fwd	TCCATGACAACTTTGGTATCGTGG
GAPDH-Rev	GACGCCTGCTTCACCACCTTCT

Table S5. Conditions for real time-PCR

**Steps**	**Conditions**
Denature	95 °C, 15s
Anealing	64 °C, 10s
Extension	72 °C, 15s

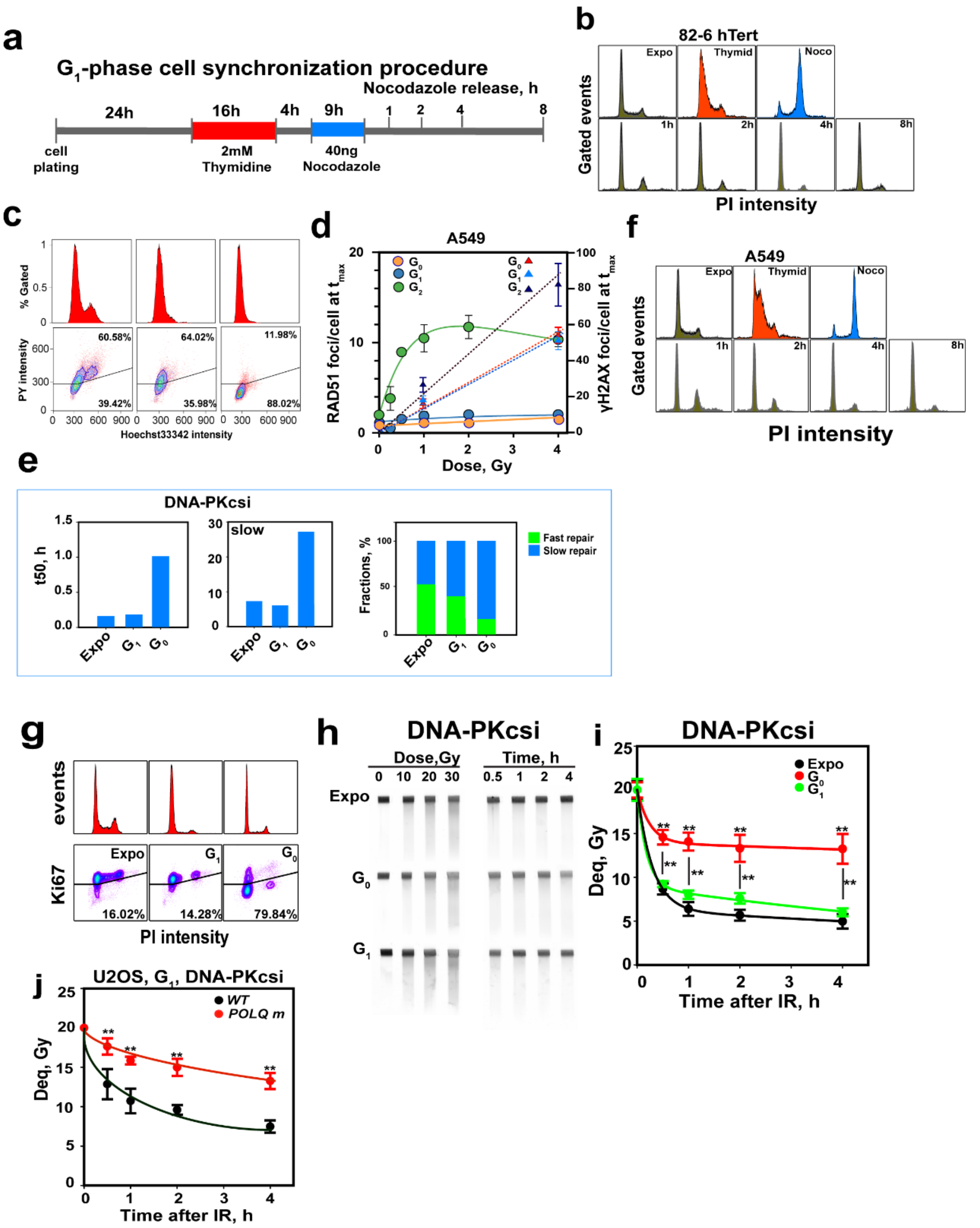

Figure S1. **Approaches for cell cycle dependent analysis.** a) Schematic representation of the G_1-phase synchronization procedure employed using thymidine combined with nocodazole treatments. Cells are allowed to grow for one division and subsequently 2 mM thymidine is added for 16 h. Cells are released from thymidine block by washing with pre-warmed PBS and are incubated for 4 h before adding nocodazole of 40 ng/mL. After 9 h of nocodazole treatment, cells are released and collected at the indicated time points to measure cell cycle distribution

and to determine a collection time depending on the desired cell cycle phase; b) Representative cell cycle distributions at different stages in the synchronization procedure outlined in a., for 82-6 hTert cells; experiments are repeated 3 times and results of a representative one are shown; c) As in Figure 1d, but for Pyronin Y staining; d) Comparison of RAD51 and γH2AX foci formation between G_0, G_1 and G_2 phase A549 cells. Cells are exposed to the indicated doses of IR and analyzed at 0.5 h, 1 h, 2 h, 4 h and 8 h later in the indicated phases of the cell cycle. Plotted is the maximum number of RAD51 (y-axis on the left) or γH2AX (y-axis on the right) foci/nucleus obtained from kinetics experiments, as a function of IR dose. The γH2AX maximum is reached at 1 h after IR independently of dose, but takes progressively longer for RAD51 with increasing dose [7]. Mean and the associated SE from 3 independent experiments are shown; e) Analysis of alt-EJ kinetics shown in Figure 1c after fitting data to the sum of two exponentials, as outlined under Materials and Methods. This fitting allows the calculation of the repair half time (t50) of the fast and slow components of rejoining, as well as the fraction of DSBs processed with fast versus slow kinetics. Specifically, A and C describe the amplitudes, while b and d the rate constants of the slow and the fast component of rejoining, respectively. Using these parameters, the half-times for the rejoining of the slow and the fast component are calculated as t50 fast = ln2/b, and t50 slow = ln2/d, respectively. The fraction of DSBs rejoined with fast kinetics is calculated as Ffast = A/(A + C) and Fslow = C/(A + C); f) As in (b) for A549 cells; g) PI and Ki67 staining for cells used in h. and i.; h) Typical images of gels from representative PFGE experiments showing the dose response, and repair kinetics in the presence of DNA-PKcsi after exposure to 20 Gy; i) Combined results from 6 determinations in two experiments such as those shown in g. Plotted is the mean and the associated SE; j) Kinetics of DSB repair after exposure to 20 Gy in the presence of DNA-PKcsi in *POLQ* wild type and mutant G_1-phase U2OS cells. Plot shows means ± SE from 6 determinations in two independent experiments.

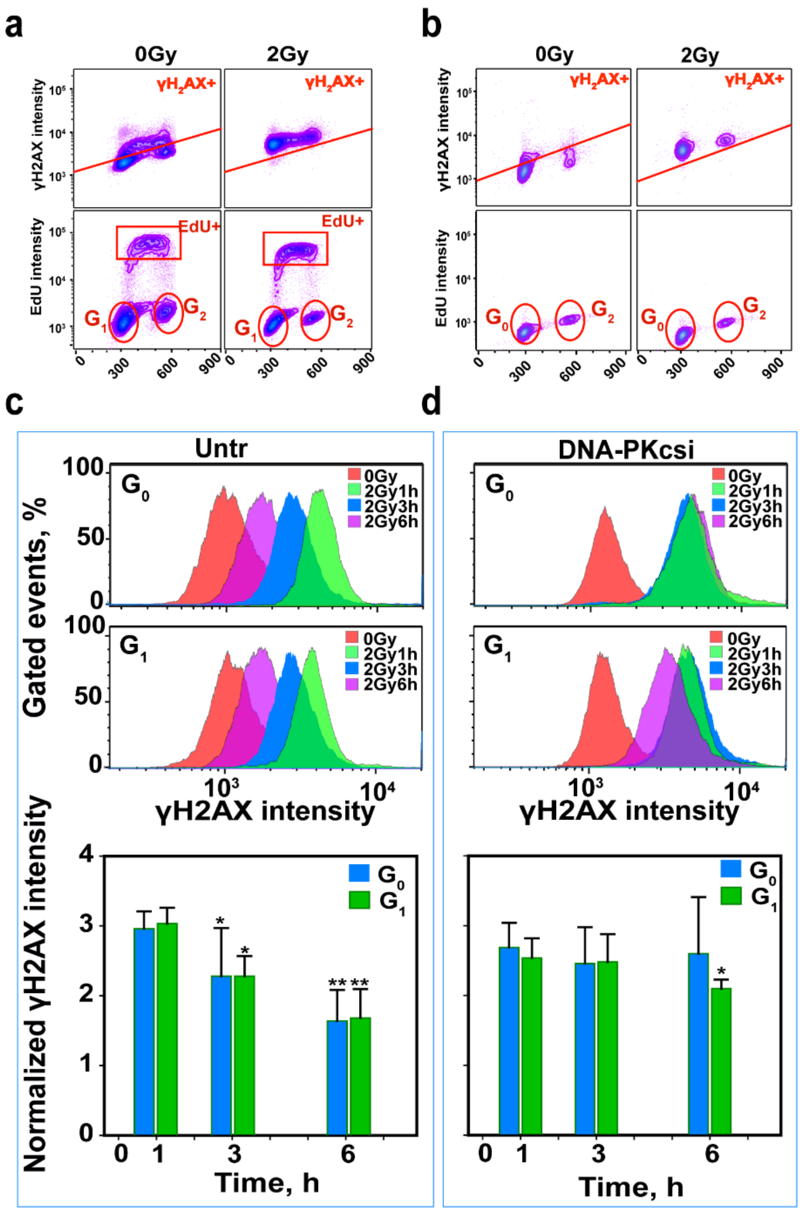

Figure S2. DSB repair analysis after 2 Gy of IR in G_1 phase 82-6 hTert cells, using γH2AX as endpoint. a) Schematic representation of the three-parametric flow cytometry analysis employed to quantitate γH2AX in cells of G_1-phase. Upper panels: Dot plots showing γH2AX versus PI signals. Lower panels: Dot plots showing EdU versus PI signals. Shown in these panels are also the gates applied for quantitating γH2AX after IR; b) As in a, but for G_0-phase cells; c) γH2AX signal decay reflecting the capacity of cells for c-NHEJ; d) γH2AX decay in the presence of DNA-PKcsi and therefore reflecting the capacity of cells for alt-EJ. Experiments are repeated 3 times and a representative is shown.

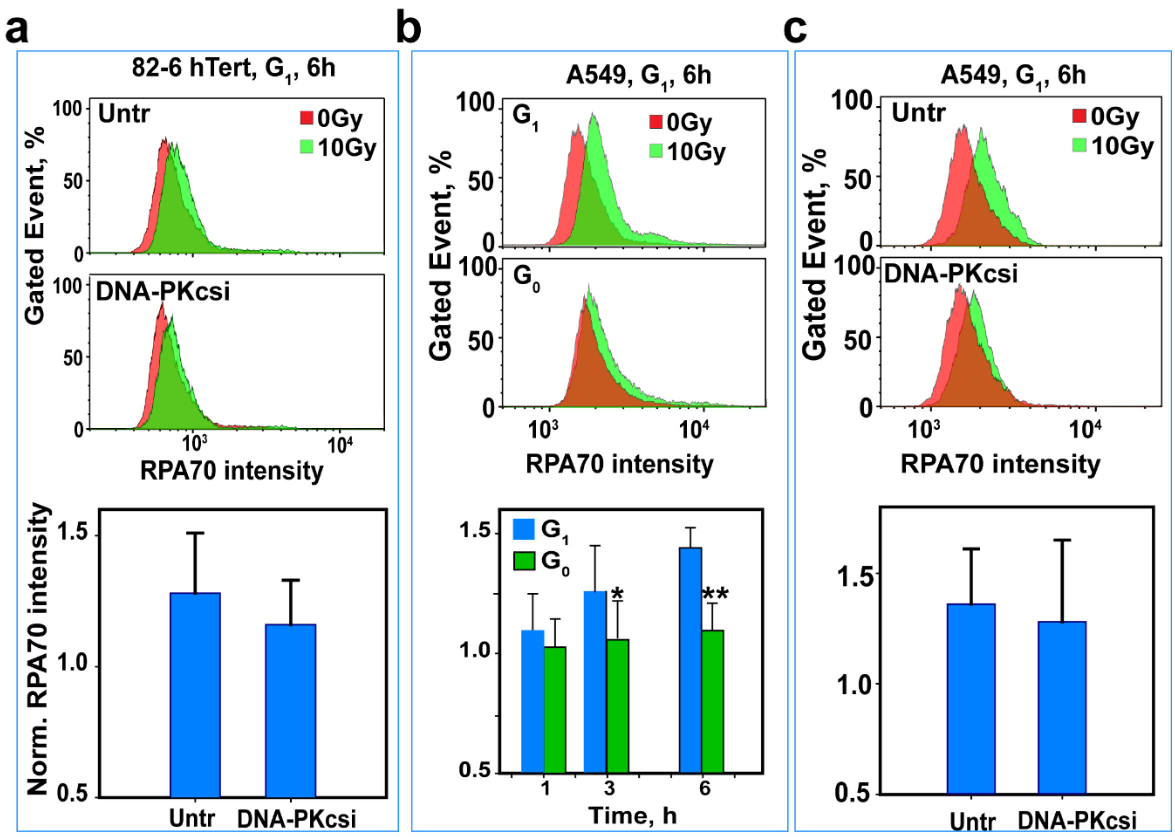

Figure S3. Resection analysis in G_0- and G_1-phase cells exposed to IR. a) Effect of DNA-PKcsi on DNA end resection measured as RPA signal intensity in G_1-phase 82-6 hTert cells 6 h after exposure to 10 Gy. Upper panel shows results of untreated cells, middle panel results of cells treated with DNA-PKcsi and the lower panel the quantitative analysis of above results using the peak values of the RPA signal distribution. b) DNA end resection in G_1- (upper panel) and G_0-phase (middle panel) A549 cells at 6 h after exposure to 10 Gy. Quantification of these results is shown in the lower panel; c) As in a. for A549 cells. Plot in the lower panels shows means ± SE from 3 independent experiments.

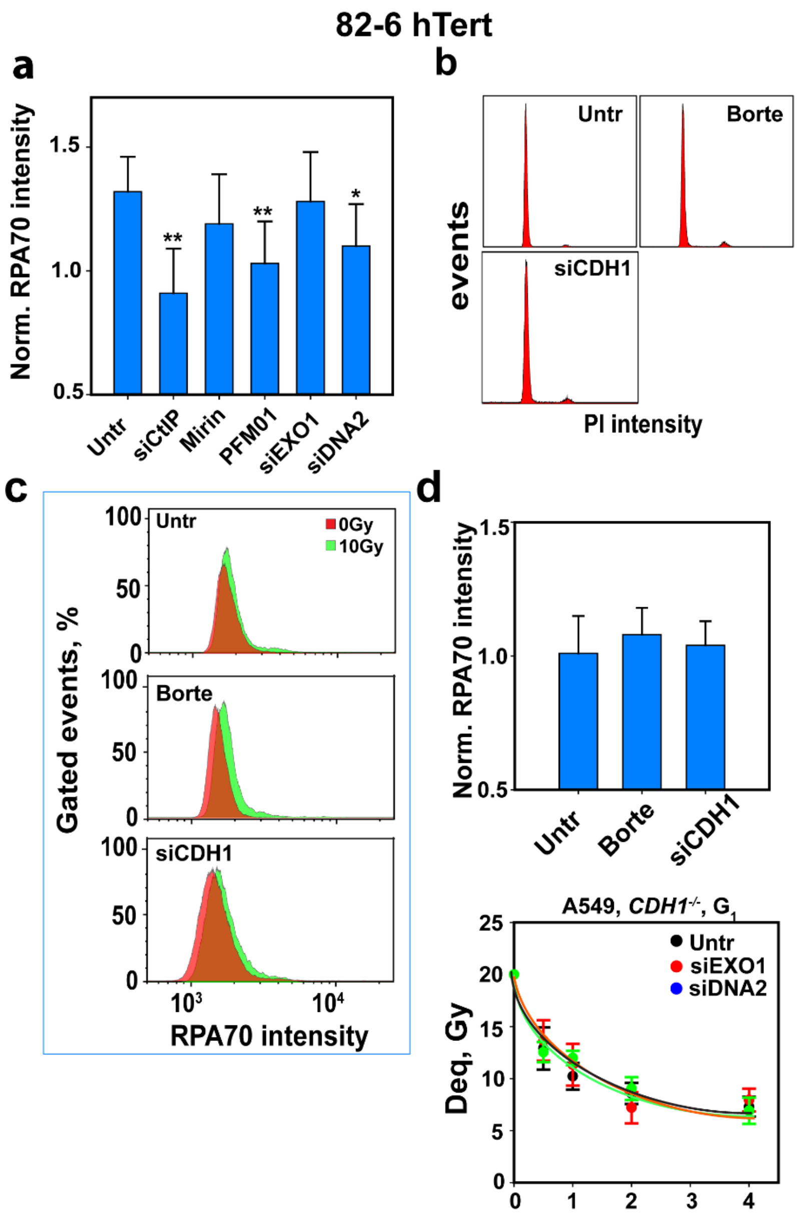

Figure S4. Effects of inhibition or knockdown of components of the resection apparatus on DNA end resection in G_1-phase 82-6 hTert cells. a) Statistical analysis of resection results shown in Figure 2g; data represent means and SE from 3 independent experiments; b) PI staining showing cell cycle distribution of cells used in Figure 3e; c) Effects of bortezomib or *CDH1* depletion on DNA end resection in G_1 phase 82-6 hTert cells; d) Quantitative analysis of results as in c. from different experiments; Plot shows means ± SE from three independent experiments; e) Effects of EXO1 or DNA2 depletion using specific siRNA, on alt-EJ in G_1-phase *CDH1*$^{-/-}$ A549 cells as determined by PFGE after exposure to 20 Gy in the presence of DNA-PKcsi. Plot shows means ±SE from 6 determinations in two independent experiments.

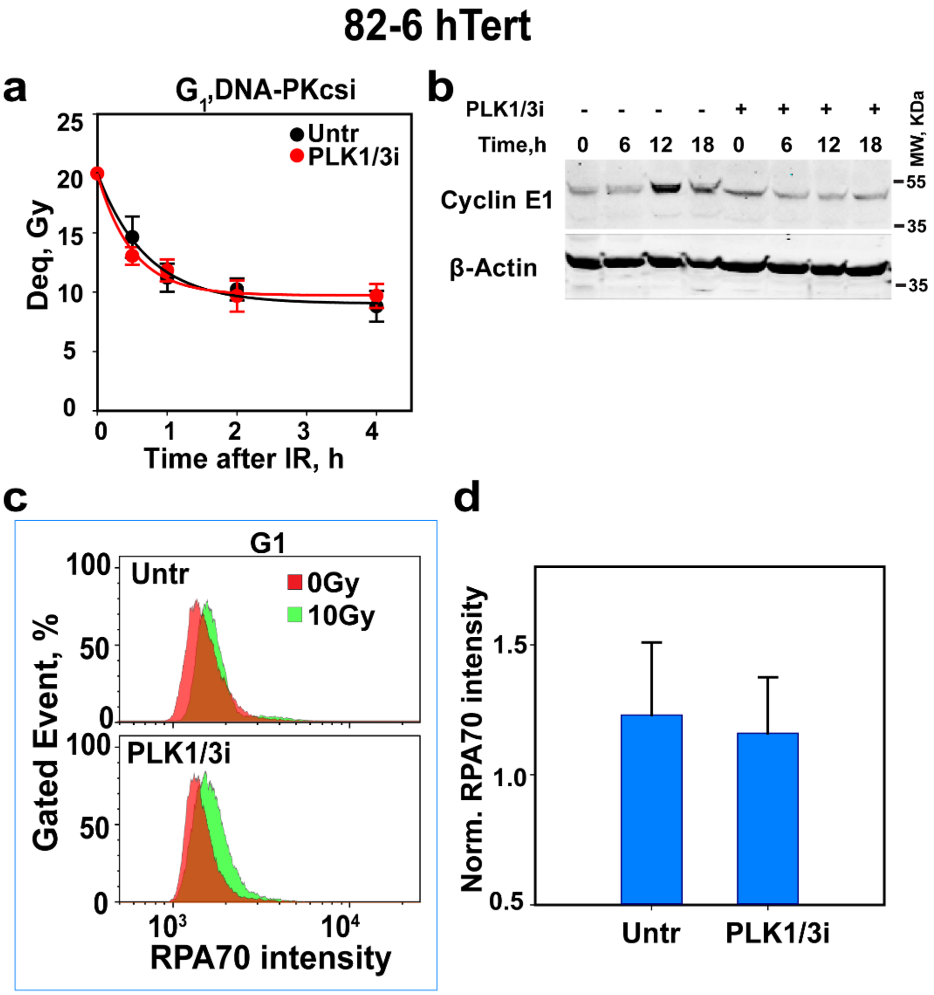

Figure S5. Effects of PLK3 inhibition on alt-EJ and resection in G_1-phase 82-6 hTert cells. a) WB analysis of cyclin E1 levels carried out to confirm the efficacy of GW843682X on PLK3 inhibition. G_0 phase 82-6 hTert cells obtained by incubation in SD-medium are stimulated to enter the cell cycle by transferring to complete growth medium in the absence or presence of 2 µM GW843682X. Cells are collected at the indicated times thereafter for WB analysis. Experiments are repeated 3 times and a representative one is shown. b) alt-EJ analysis using PFGE of G_1-phase 82-6 hTert cells after exposure to 20 Gy and inhibition of PLK3; data represent means and SE of 6 determinations from two experiments; c) Resection analysis by quantification of RPA70 signal intensity after treatment with a PLK1/3 inhibitor; d) Quantification of RPA70 signal intensity for results like those shown in b. Plot shows means ± SE from 3 independent experiments.

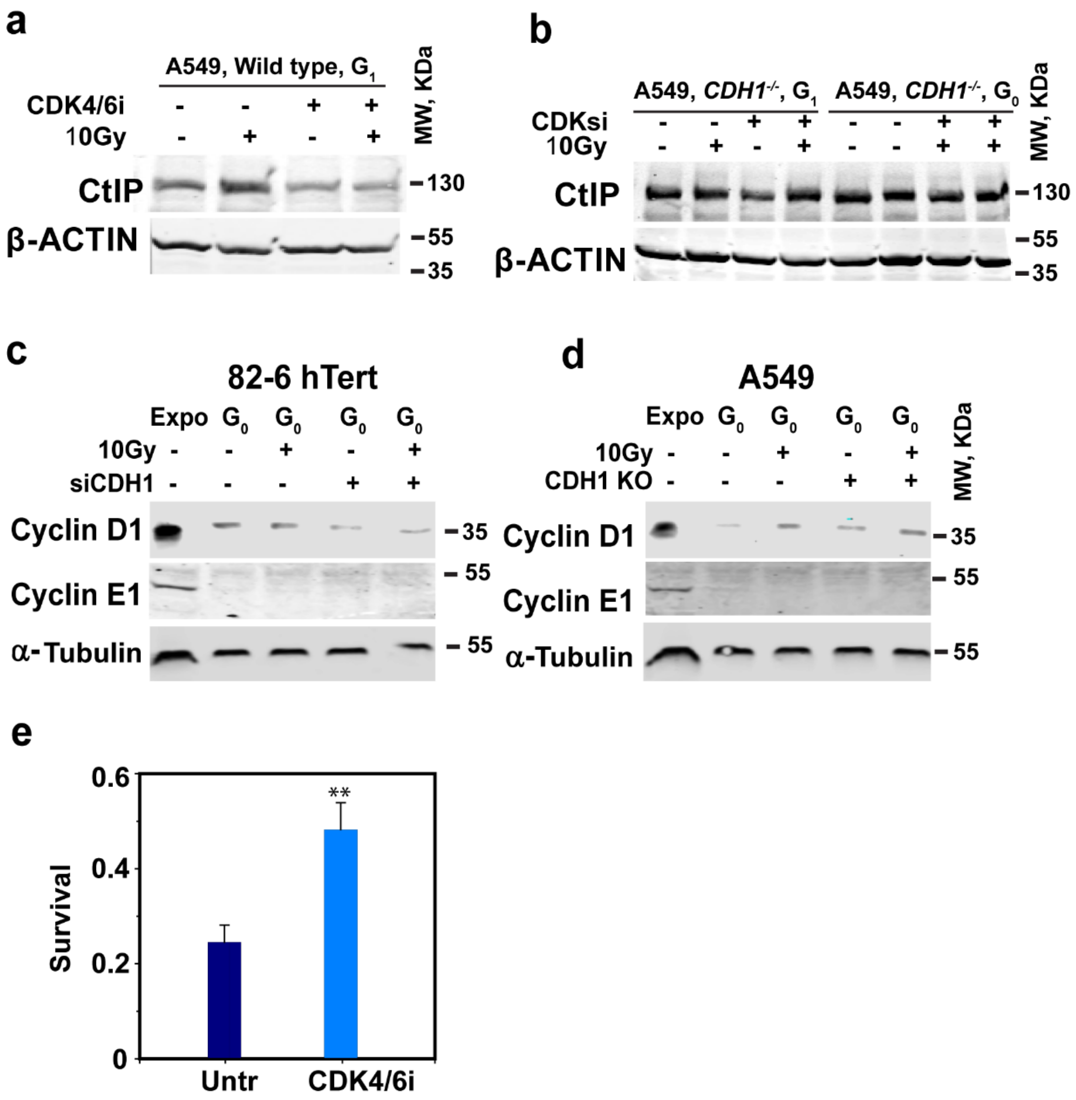

Figure S6. Effects of CDKs on CtIP level in *CDH1* wild type and *CDH1* deficient cells. a) WB-analysis of the effect of CDK4/6 inhibition on CtIP level after exposure to 0 Gy or 10 Gy in G_1 phase A549 cells; b) WB-analysis of the effects of combined CDKs inhibition on CtIP level after exposure to 0 Gy or 10 Gy in G_1 or G_0 phase $CDH1^{-/-}$ A549 cells; c) WB-analysis of *CDH1* depletion on cyclin D1 and E1 in A549 cells; d) As in c. for 82-6 hTert cells; e) Effects of CDK4/6i on cell radiosensitivity to killing after exposure to 4 Gy in G_1 phase A549 cells, determined by colony formation as described in Material and Methods. Plot shows means ± SE from 3 independent experiments. Experiments in a. -d. are repeated 3 times and a representative one is shown.

Supplementary References

1. Barton, O.; Naumann, S.C.; Diemer-Biehs, R.; Künzel, J.; Steinlage, M.; Conrad, S.; Makharashvili, N.; Wang, J.; Feng, L.; Lopez, B.S.; et al. Polo-like kinase 3 regulates CtIP during DNA double-strand break repair in G1. *Journal of Cell Biology* **2014**, *206*, 877-894, doi:10.1083/jcb.201401146.
2. Lafranchi, L.; de Boer, H.R.; de Vries, E.G.; Ong, S.E.; Sartori, A.A.; van Vugt, M.A. APC/CCdh1 controls CtIP stability during the cell cycle and in response to DNA damage. *EMBO Journal* **2014**, *33*, 2860-2879, doi:10.15252/embj.201489017.
3. Tomimatsu, N.; Mukherjee, B.; Catherine Hardebeck, M.; Ilcheva, M.; Vanessa Camacho, C.; Louise Harris, J.; Porteus, M.; Llorente, B.; Khanna, K.K.; Burma, S. Phosphorylation of EXO1 by CDKs 1 and 2 regulates DNA end resection and repair pathway choice. *Nature Communications* **2014**, *5*, 3561, doi:10.1038/ncomms4561.
4. Wu, X.; Wang, B. Abraxas suppresses DNA end resection and limits break-induced replication by controlling SLX4/MUS81 chromatin loading in response to TOP1 inhibitor-induced DNA damage. *Nat Commun* **2021**, *12*, 4373, doi:10.1038/s41467-021-24665-w.
5. Elbashir, S.M.; Harborth, J.; Lendeckel, W.; Yalcin, A.; Weber, K.; Tuschl, T. Duplexes of 21-nucleotide RNAs mediate RNA interference in cultured mammalian cells. *Nature* **2001**, *411*, 494-498, doi:10.1038/35078107.
6. Kenny, M.K.; Schlegel, U.; Furneaux, H.; Hurwitz, J. The role of human single-stranded DNA binding protein and its individual subunits in simian virus 40 DNA replication. *Journal of Biological Chemistry* **1990**, *265*, 7693-7700.
7. Li, F.; Mladenov, E.; Mortoga, S.; Iliakis, G. SCF(SKP2) regulates APC/C(CDH1)-mediated degradation of CTIP to adjust DNA-end resection in G2-phase. *Cell Death Dis* **2020**, *11*, 548, doi:10.1038/s41419-020-02755-9.
